# Bridging the implementation gap in cardiovascular prevention: a narrative review and call to action

**DOI:** 10.3389/ijph.2026.1609454

**Published:** 2026-06-10

**Authors:** Grzegorz Kubielas, Jacek Gonos, Christopher S. Lee, Angela Durante, Michela Barisone, Nicola Straiton, Adrian Jurczyk, Katarzyna Kułaga, Maria Jędrzejczyk, Izabella Uchmanowicz

**Affiliations:** 1 Department of Healthcare Organization, Department of Nursing, Faculty of Nursing and Midwifery, Wroclaw Medical University, Wrocław, Poland; 2 Department of Healthcare Services, Polish National Health Fund, Central Office in Warsaw, Warsaw, Poland; 3 Foundation of Wroclaw Medical University, Wroclaw, Poland; 4 Boston College William F. Connell School of Nursing, Chestnut Hill, MA, United States; 5 Division of Scientific Research and Innovation in Emergency Medical Service, Department of Emergency Medical Service, Faculty of Nursing and Midwifery, Wroclaw Medical University, Wroclaw, Poland; 6 Health Science Interdisciplinary Center, Sant’Anna School of Advanced Studies, Pisa, Italy; 7 Nurse Research Manager Fondazione Toscana Gabriele Monasterio Via Moruzzi 1, Pisa, Italy; 8 Clinical Epidemiology and Research Center, IRCCS Humanitas Research Hospital, Rozzano, Italy; 9 School of Nursing, Midwifery and Paramedicine, Australian Catholic University, North Sydney, NSW, Australia; 10 Nursing Research Institute, St Vincent’s Health Network Sydney St Vincent’s Hospital Melbourne, Australian Catholic University, Darlinghurst, NSW, Australia; 11 Division of Research Methodology, Department of Nursing, Faculty of Nursing and Midwifery, Wroclaw Medical University, Wrocław, Poland; 12 Centre for Cardiovascular Health, Edinburgh Napier University, Edinburgh, United Kingdom

**Keywords:** cardiovascular disease prevention, community health programmes, health systems, implementation science, primary prevention

## Abstract

**Objectives:**

Cardiovascular disease (CVD) remains a leading cause of morbidity and mortality worldwide, yet substantial gaps persist between evidence-based prevention strategies and their real-world implementation. This narrative review aimed to identify and synthesize contemporary models, programmes, and implementation strategies in preventive cardiovascular care, highlighting factors that facilitate or hinder adoption at scale.

**Methods:**

Narrative synthesis drawing on searches in MEDLINE (PubMed), Embase, and the Cochrane Library (to July 2025), prioritising systematic reviews, major guideline statements, and large multicentre studies.

**Results:**

Community programmes yield modest but meaningful reductions in blood pressure, lipids, and glucose. Clinical programmes achieve greater individual-level effects but are constrained by limited reach. Key barriers include misaligned incentives, workforce limitations, and persistent inequities. The Consolidated Framework for Implementation Research (CFIR) and Reach, Effectiveness, Adoption, Implementation, Maintenance (RE-AIM) frameworks remain underutilised. Emerging digital tools and updated cardiovascular risk models offer new opportunities but require pragmatic integration.

**Conclusion:**

Strengthening preventive cardiovascular care requires aligning health-system incentives, integrating implementation science, and leveraging technology to support scalable and equitable prevention models.

## Introduction

Cardiovascular disease (CVD) continues to impose a major global health and economic burden, despite decades of advances in prevention and treatment. Each year, CVD causes over 20 million deaths worldwide and exacts substantial costs on healthcare systems and societies [1–3]. Importantly, a large proportion of CVD is attributable to modifiable risk factors, and many cases are preventable through evidence-based interventions [4, 5].

Over recent decades the focus in cardiovascular medicine has shifted from acute care and event-driven secondary prevention towards broader primary-prevention strategies that address the upstream drivers of atherosclerotic disease [6–10]. Modern prevention programmes are therefore increasingly multi-component, targeting behavioural, metabolic, environmental and social determinants of risk rather than single isolated factors.

Although the evidence base for many preventive interventions is strong, routine implementation remains inconsistent. The so-called 60-30-10 challenge summarises this problem: roughly 60% of care aligns with best evidence, about 30% is low-value or wasteful, and approximately 10% may be harmful [11]. Moreover, there is evidence that it can take up to 17 years for robust research findings to be routinely implemented in clinical practice, and fewer than one in five evidence-based interventions are ever widely adopted [12]. These figures underscore that the central challenge is not simply generating evidence but ensuring its translation into everyday care. Implementation science offers concepts and tools to understand barriers and to guide adoption, yet its methods remain under-utilised in many practice settings [13].

This review summarises the evidence for community-based and clinical prevention programmes, examines implementation gaps, and discusses how emerging technologies and system reforms could improve delivery and upscaling of effective interventions.

## Methods

This paper is not a systematic review and does not follow PRISMA methodology. Instead, it is a structured discussion paper that integrates key evidence sources and contextualises them with reference to implementation challenges, economic considerations and technological opportunities, with a focus on lessons applicable to diverse European healthcare systems.

Findings from systematic reviews, guideline documents, large prospective studies, registry data and exemplar prevention programmes were synthesised. Searches were conducted in MEDLINE (PubMed), Embase and the Cochrane Library for relevant literature published up to July 2025. Priority was given to high-quality reviews, major guideline statements and large multicentre studies (such as EUROASPIRE and comprehensive clinical programme evaluations). Evidence was selected to highlight representative findings and to illustrate common challenges and strategies in cardiovascular prevention.

The evidence base was assembled using two complementary approaches. First, systematic database searches were conducted in MEDLINE (PubMed), Embase, and the Cochrane Library using the following search concept clusters: (1) cardiovascular disease prevention programmes (community-based cardiovascular prevention, clinical cardiovascular prevention, cardiac rehabilitation, secondary prevention cardiology, preventive cardiology programmes); (2) implementation science and health systems (implementation science, CFIR, RE-AIM, implementation gap, health system reform, value-based care); (3) surveillance and registries (EUROASPIRE, cardiovascular prevention registry); and (4) economic evaluation (cost-effectiveness cardiovascular prevention, economic burden cardiovascular disease, return on investment prevention). The search covered publications up to July 2025. Inclusion criteria for this category were: systematic reviews, large multicentre trials, registry-based studies, and clinical programme evaluations reporting on programme design, effectiveness, adoption, or implementation in primary or secondary cardiovascular prevention in adults; written in English. Conference abstracts without full text and single case reports were excluded. Exemplar programmes were selected on the basis of methodological quality, programme comprehensiveness, availability of implementation and outcome data, and geographic representativeness (preference given to European or multi-country data). Second, targeted citation of background and technical references was used for three additional categories of evidence that do not arise from programme-level searches but are necessary to contextualise findings: (a) epidemiological burden estimates and guideline documents; (b) meta-analyses of specific risk factor interventions (blood pressure, lipid-lowering, Mediterranean diet, physical activity, sleep, smoking cessation) cited to quantify the clinical significance of observed programme effects; and (c) validated assessment instruments cited within exemplar programme descriptions. The selection process carries an inherent risk of selective citation bias, which is acknowledged as a limitation of the narrative review design.

## Results

### The evidence base for community-based prevention

The foundation for understanding the effectiveness of cardiovascular prevention programmes has been substantially strengthened by large-scale systematic reviews that examine the collective impact of community-based interventions across diverse populations and settings. Community-based interventions are generally defined as health strategies delivered outside traditional clinical environments – for example in schools, workplaces, neighbourhoods or faith-based organisations – that engage local resources and stakeholders to promote cardiovascular health and reduce risk factors at the population level [14]. According to the World Health Organization, such interventions seek to “empower communities through participatory approaches to improve health outcomes and reduce health inequities” [15].

One comprehensive synthesis by Soltani and colleagues examined 48 community-based cardiovascular prevention programmes and provides a broad evidence base on programme effectiveness [16]. The review screened nearly 12,000 titles and abstracts before including studies that met strict quality and outcome-reporting criteria, increasing confidence that the pooled results reflect robust evidence [16]. Overall, the meta-analysis reported modest but meaningful population-level improvements. Participating caommunities experienced average reductions of 2.90 mmHg in systolic blood pressure and 2.21 mmHg in diastolic blood pressure. Although these changes are numerically modest, their public-health impact is considerable: small shifts in population blood pressure are associated with substantial reductions in stroke and myocardial infarction incidence. Epidemiological evidence supports the conclusion that even modest average reductions in blood pressure can translate into thousands of prevented cardiovascular events across populations [17, 18]. Community programmes also delivered favourable changes in lipid profiles. Low-density lipoprotein cholesterol (LDL-C) decreased by an average of 8.88 mg/dL, and triglycerides fell by 8.40 mg/dL, with corresponding reductions in total cholesterol; high-density lipoprotein cholesterol (HDL-C) showed no consistent change. This pattern is consistent with lifestyle interventions that preferentially improve atherogenic lipid fractions while producing only modest effects on HDL-C [19, 20]. Modest but consistent improvements in fasting blood glucose were observed across included programmes. Although the glucose reductions were smaller than those for blood pressure and lipids, the consistency of the effect across diverse interventions suggests that community-based strategies can favourably influence glucose metabolism at the population level [21].

In the Soltani analysis, programme heterogeneity means effects were not uniform [16]. Comparative analysis of programme characteristics indicates that the most successful interventions shared several features. Effective programmes typically targeted multiple interacting risk factors rather than single isolated behaviours. Strong community engagement – including mobilisation of local organisations, schools, workplaces and primary-care services – was repeatedly associated with broader reach and greater sustainability. Environmental and policy actions (for example, improved access to healthy foods, smoke-free policies and safe spaces for physical activity) further amplified individual-level behaviour change by changing the contexts in which choices are made.

Community engagement emerged as a crucial success factor in the Soltani analysis [16]. Programmes that mobilised local organisations, schools, workplaces, and healthcare services generally achieved greater reach and more sustained impact than those relying solely on provider-initiated approaches. This suggests that sustainable improvements in cardiovascular health require broad-based social engagement as well as clinical action. Notably, several successful programmes explicitly applied implementation-science frameworks to guide design, evaluation and scale-up: CFIR helped identify multilevel barriers and facilitators, RE-AIM supported assessment of reach, adoption, implementation and maintenance, and process models such as PRISM and EPIS assisted in planning phased implementation and sustainment. Using these frameworks appears to strengthen the translation of community engagement into durable outcomes by systematising context adaptation and monitoring of implementation metrics (e.g. reach, fidelity, maintenance) [22–25].

### Clinical prevention programmes: lessons from the cardiovascular health program registry

While community-based programmes provide insights into population-level prevention strategies, comprehensive clinical prevention programmes offer a different perspective on what can be achieved through intensive, individualised interventions. The Cardiovascular Health Program Registry represents one of the most thoroughly studied examples of this approach, providing detailed insights into both the potential and the challenges of clinic-based cardiovascular prevention [26].

### Cardiovascular health program registry

This prospective study followed nearly 1,000 participants through a comprehensive 12-month therapeutic-lifestyle-change programme that embodied many principles considered best practice in cardiovascular prevention. The programme was built around the investigators’ “four pillars of optimal health”: dietary optimisation, physical-activity enhancement, stress management, and sleep improvement. This comprehensive approach reflects the recognition that cardiovascular health depends on multiple, interconnected lifestyle factors that must be addressed simultaneously for optimal results [26, 27].

The dietary component in the Cardiovascular Health Program Registry emphasised Mediterranean-style eating patterns, which have substantial evidence for cardiovascular benefit [28, 29]. Participants received practical guidance on increasing whole grains, vegetables, fruits and nuts, while limiting red meat and moderating sodium and saturated fat intake [26]. The emphasis was on sustainable dietary patterns rather than short-term restrictive diets. Physical activity recommendations balanced aerobic and strength-training exercises and aligned with established guidance advocating at least 150 min of moderate-intensity activity per week plus regular muscle-strengthening sessions [30]. Recognising that long-term adherence depends on enjoyment and feasibility, participants were encouraged to select personally rewarding activities that fitted their daily lives [26].

To support sustained behaviour change, successful programmes routinely integrated explicit implementation strategies [31]. These included evidence-based behaviour-change techniques (goal-setting, action planning and self-monitoring), motivational interviewing or brief counselling to strengthen intrinsic motivation, peer-led groups and local “community champions” to bolster social support and local ownership, and alignment with environmental or policy interventions (for example, activity-friendly routes and workplace schemes) to reduce structural barriers [32]. Many projects selected and reported implementation strategies using taxonomies such as ERIC, and monitored implementation outcomes with frameworks like RE-AIM and CFIR to measure reach, fidelity, adoption and sustainment and to allow adaptive improvements in real time [33–35].

Stress-management components incorporated evidence-based techniques such as guided imagery, diaphragmatic breathing and progressive-muscle relaxation. Participants also learned a brief stress-reduction exercise (the “tension tamer”) intended for use during acute stress episodes. This pragmatic approach aligned stress management with daily routines rather than treating it as an isolated activity [36].

Sleep optimisation was a particularly novel element of the programme, reflecting growing recognition of sleep as an important determinant of cardiovascular health. Participants received individualised sleep-hygiene interventions based on cognitive-behavioural principles together with environmental modifications designed to improve sleep quality and duration [26, 37, 38].

The programme used validated assessment tools to measure behavioural and clinical changes, including the Rate-Your-Plate dietary questionnaire [39], the International Physical Activity Questionnaire [40], the Perceived Stress Scale [41], the Pittsburgh Sleep Quality Index [42], the Epworth Sleepiness Scale [43], and the Berlin Questionnaire for sleep-apnoea risk [44]. These standardised instruments enabled systematic evaluation of lifestyle behaviours and their change over the intervention period.

Outcomes were notable. Behavioural changes achieved during the 12-month programme were both large and sustained. The proportion of participants meeting predefined dietary targets increased from 53% at baseline to 86% at 12 months, while adherence to physical-activity recommendations rose from 44% to 66% (mean increase >70 min of activity per week). Perceived stress scores declined substantially, and the share of participants meeting stress-management goals increased from 65% to 79%. Sleep quality also improved markedly: the fraction of participants meeting sleep-quality targets rose from 28% to 49%, together with clinically meaningful increases in average sleep duration [26].

These behavioural gains translated into clinically meaningful risk-factor changes. Among participants with elevated blood pressure at baseline, 69% experienced reductions in systolic blood pressure and 71% had reductions in diastolic blood pressure. Lipid outcomes were similarly encouraging: 74% of individuals with raised total cholesterol improved, 65% with elevated LDL-C improved, and 86% with elevated triglycerides achieved reductions. Improvements in weight and body composition were observed as well: 63% of overweight/obese participants reduced BMI, while central obesity improved in 71% of men and 74% of women with elevated waist circumference. Metabolic markers improved too: 72% of participants with elevated fasting glucose showed reductions and 71% of those with insulin resistance demonstrated better glycaemic control [26].

Importantly, most improvements occurred with minimal medication changes: the majority of participants had no adjustments to antihypertensive, lipid-lowering or antidiabetic regimens during follow-up, and a pre-specified sub-analysis of those without medication changes indicated that lifestyle modification was the principal contributor to the observed risk-factor reductions. This observation supports the potential of well-implemented comprehensive lifestyle programmes to effect clinically relevant change independent of pharmacotherapy [26].

### The implementation reality: lessons from EUROASPIRE

While research studies demonstrate what can be achieved under optimal conditions, understanding the real-world implementation of cardiovascular prevention requires examination of how evidence is translated into routine clinical practice. The EUROASPIRE surveys, conducted across multiple European countries over several decades, provide unparalleled insights into persistent gaps between evidence-based recommendations, guidelines and actual clinical practice [45–49]. The EUROASPIRE IV survey, the most recent and comprehensive of these assessments, examined both secondary prevention practices among patients with established coronary disease and primary-prevention approaches in individuals at high cardiovascular risk. The findings paint a sobering picture of implementation challenges that persist despite decades of guideline development and dissemination efforts [45].

Among patients with established coronary disease in EUROASPIRE IV, lifestyle risk factors remain disturbingly prevalent despite their clear implications for prognosis. In the study cohort, 16% of patients continued to smoke, and only about half of those who were smoking at the time of their index cardiac event had quit by follow-up. This is particularly concerning because smoking cessation after myocardial infarction or diagnosis of coronary heart disease is associated with large reductions in mortality and recurrent events – pooled analyses estimate roughly a one-third reduction in all-cause mortality for quitters compared with persistent smokers [50]. Large-scale surveillance studies echo these findings, showing persistently high rates of smoking among coronary patients across settings and underlining the need for improved secondary-prevention strategies [51]. To improve prognosis, cardiac secondary-prevention programmes should therefore prioritise systematic identification of current smokers and the routine offer of evidence-based cessation support. Effective approaches include brief inpatient counselling with organised follow-up after discharge, behavioural support (individual or group), and pharmacotherapy (nicotine replacement therapy, bupropion, or varenicline), ideally used in combination and delivered with longitudinal follow-up; these strategies have been shown to increase quit rates in cardiac populations and are safe from a cardiovascular perspective [52–54]. Implementing these measures at scale will require integration of cessation protocols into hospital discharge pathways and community follow-up, training of clinical teams in delivery and referral, and routine monitoring of smoking status as a quality metric for secondary-prevention programmes [51].

Physical inactivity was even more prevalent, with 60% of coronary patients reporting little or no regular physical activity in EUROASPIRE IV. This finding is especially troubling given robust evidence for exercise benefits in secondary prevention and the availability of cardiac rehabilitation programmes specifically designed to address this issue. The persistence of physical inactivity among coronary patients suggests fundamental challenges in current approaches to lifestyle counselling and support. Obesity rates among coronary patients were alarmingly high, with 38% meeting criteria for obesity and 58% exhibiting central obesity patterns particularly associated with cardiovascular risk. These observations suggest that the acute cardiac event – which might be expected to motivate lifestyle change – is often insufficient to produce sustained weight management without comprehensive support programmes.

Risk-factor control, even with appropriate medications, showed concerning deficiencies across multiple parameters among participants in EUROASPIRE IV. Blood pressure control was suboptimal in 43% of coronary patients despite widespread antihypertensive use. Even more striking, 81% of patients had LDL cholesterol above the guideline-recommended target of 1.8 mmol/L (70 mg/dL), despite 86% being prescribed statin therapy. Glycaemic control among diabetic coronary patients was similarly disappointing, with only 53% achieving an HbA1c < 7.0%. This is particularly important given the established benefits of glycaemic control in people with coronary disease and the availability of effective glucose-lowering therapies.

Primary-prevention findings were likewise concerning in EUROASPIRE IV, revealing substantial missed opportunities for risk reduction in high-risk individuals. Among people at high cardiovascular risk due to hypertension, dyslipidaemia or diabetes but without established cardiovascular disease, 17% continued to smoke, 44% were obese, and 64% exhibited central obesity. Risk-factor control in this high-risk group mirrored the deficiencies seen in secondary prevention: only 43% of individuals on antihypertensive therapy achieved target blood pressure, and just 33% of those on lipid-lowering therapy reached LDL-C goals. Among high-risk individuals with diabetes, 59% achieved glycaemic targets – better than in the secondary-prevention group but still leaving considerable scope for improvement.

These findings from routine clinical practice stand in stark contrast to the results achieved in research settings, highlighting the substantial implementation gap that persists in cardiovascular prevention. Cardiac rehabilitation (CR) is associated with reduced mortality and recurrent events; real-world data show that patients who complete CR have substantially lower 12-month mortality and cardiovascular readmission rates [55]. Whether these benefits are realised broadly in routine care is uncertain, however, because participation remains low: global reports indicate that only around 20%–50% of eligible patients attend CR programmes, with wide variation between settings [56]. Low referral and participation rates are driven largely by system-level deficits – absence of systematic or automatic referral pathways, inconsistent physician endorsement, and lack of standardised administrative and e-referral processes – together with well-documented patient-level barriers (distance, cost, work commitments and socio-demographic factors). Addressing these structural and process barriers (for example, via automatic referral, e-referral systems, telehealth options and targeted quality-improvement initiatives) is therefore critical if CR’s proven benefits are to be realised at scale [57].

The EUROASPIRE surveys illustrate that, although effective interventions for cardiovascular risk reduction exist, they are frequently not implemented consistently in routine care across Europe. EUROASPIRE is a series of large multicentre surveys conducted under the auspices of the European Society of Cardiology and involving between 15 and 27 European countries across different survey waves; these surveys repeatedly document suboptimal lifestyle and risk-factor control despite high medication use [45, 46, 48, 58]. Crucially, this shortfall often reflects failures to apply established implementation-science methods and to collaborate with implementation scientists, rather than a lack of effective clinical interventions *per se*. Implementation science offers theory-based frameworks and discrete implementation strategies to identify context-specific barriers, select tailored strategies and monitor implementation outcomes (e.g. reach, fidelity, adoption, maintenance); yet these approaches remain under-used in many real-world prevention programmes. Integrating implementation science into programme design, evaluation and scale-up is therefore essential if evidence-based cardiovascular interventions are to be translated reliably into routine practice [13, 58].

Temporal trends observed across multiple EUROASPIRE surveys reveal both encouraging and concerning patterns. Improvements in blood-pressure and lipid control over the past two decades reflect advances in pharmacotherapy, clinical guidelines and increased clinical attention to these risk factors. However, these gains have been counterbalanced by rising prevalences of obesity, diabetes and physical inactivity, indicating that health systems are successfully addressing some biomedical risk factors while failing to prevent upstream determinants of cardiometabolic disease. For clarity, these trends principally reflect performance within the European healthcare systems that participated in EUROASPIRE – a heterogeneous mix of predominantly high-income countries with national health services, social-insurance models and mixed public–private arrangements – and encompass outcomes measured across primary care, hospital-based secondary-prevention services and community/public-health programmes. This pattern suggests that while clinical care pathways and pharmacological management have improved, broader population-level prevention (shaped by public-health policy, the built environment and social determinants) remains inadequately addressed in many participating systems [59, 60].

To allow direct comparison of the two major programme types discussed, Table 1 summarises community-based and clinical prevention programmes across five RE-AIM domains (Reach, Effectiveness, Adoption, Implementation, and Maintenance), and provides indicative information on resources and equity impact. This structured comparison is intended to help readers identify context-appropriate programme designs and to facilitate adaptation at local level (Table 1).

**TABLE 1:** Comparison of Community-Based and Clinical Cardiovascular Prevention Programmes Using RE-AIM Domains (European countries, 1997–2024).

Domain	Community-based programmes	Clinical prevention programmes
Reach	Broad; targets general or high-risk populations in non-clinical settings	Narrower; reaches patients who access or are referred to clinical services
Effectiveness	Modest but meaningful population-level reductions in BP, LDL-C, and glucose	Larger individual-level effect sizes; significant risk factor improvements at 12 months
Adoption	Highly variable; depends on community trust, cultural fit, and stakeholder engagement	Constrained by referral gaps; ∼20–50% of eligible patients attend cardiac rehabilitation
Implementation	Fidelity varies; benefits from structured frameworks (CFIR, RE-AIM) and trained lay facilitators	Typically multidisciplinary; requires trained clinical staff, validated tools, and structured protocols
Maintenance	Sustained when embedded in local structures; vulnerable to funding cycles	Requires ongoing institutional support; long-term adherence is a known challenge
Resources (staff, cost)	Lower *per capita* cost; relies on community health workers and volunteers; scalable	Higher per-participant cost; requires trained clinicians and dedicated facilities
Equity impact	Greater potential to reach deprived or underserved populations when culturally tailored	Risk of widening inequalities if access barriers (distance, cost, referral) are not addressed

## Discussion

### Emerging technologies and future directions

The landscape of cardiovascular prevention is being rapidly transformed by technological innovations that create new possibilities for risk assessment, intervention delivery and outcome monitoring. These emerging technologies hold promise for addressing some of the implementation challenges that have limited the effectiveness of traditional prevention approaches. At the same time, they create an “innovation challenge”: many digital and technology-enabled interventions remain confined to pilot testing and fail to reach routine care at scale. Addressing this risk requires pairing innovation with implementation-focused strategies such as co-design with end users, pragmatic evaluation, and clear plans for integration and sustainability [61]. A summary of key innovations, their implementation challenges, and opportunities for practice and research is presented in Table 2.

**TABLE 2:** Emerging technologies and future directions in cardiovascular disease prevention.

Domain/Technology	Key advances	Implementation challenges	Opportunities for practice and research
Risk prediction models	SCORE2/SCORE2-OP/SCORE2-Diabetes incorporate contemporary data and non-fatal events, offering population-tailored risk estimates	Need for validation in diverse populations; updating data inputs over time	Enhance precision in primary prevention and align treatment intensity with risk
Artificial intelligence (AI)	Machine-learning models improve risk prediction and identify complex data patterns	Limited external validation; transparency and interpretability issues	Personalised prevention strategies; integration into clinical decision support
Digital health tools (mHealth, wearables)	Enable real-time lifestyle feedback and continuous physiological monitoring	Variable accuracy; few tools certified as medical devices; regulatory uncertainty	Support patient engagement, remote monitoring and sustained behaviour change
Telemedicine platforms	Expand access to prevention services and support longitudinal monitoring	Digital divide; workflow integration; reimbursement models	Improve equity and continuity of care, especially post-COVID-19
Advanced imaging	CAC scoring provides direct assessment of atherosclerotic burden for better risk stratification	Cost, radiation exposure, and limited accessibility in some settings	Refine preventive therapy decisions in intermediate-risk patients
Precision medicine/Genomics	Polygenic risk scores improve stratification beyond traditional factors	High cost; uncertain clinical utility; equity concerns	Potential to personalise prevention when combined with clinical and lifestyle data
Biomarkers	Novel markers (e.g. Lp(a), inflammatory and proteomic indicators) refine risk estimation	Need for standardisation, cost-effectiveness data, and outcome validation	Guide tailored interventions and early identification of high-risk individuals

Risk assessment tools have evolved beyond traditional approaches, with new calculators offering more sophisticated and personalised risk prediction. The SCORE2 and SCORE2-OP systems represent advances by incorporating contemporary epidemiological data and providing tailored estimates for different age groups and regions [6, 62]. Specialised tools such as SCORE2-Diabetes further reflect the need for population-specific risk assessment, integrating predictors such as diabetes duration, glycaemic control and renal function [62]. Cardiac rehabilitation programmes remain a key element of secondary prevention, yet EUROASPIRE data indicate that only around half of eligible coronary patients are referred, representing a major missed opportunity for improving outcomes [45, 63–65].

Artificial intelligence (AI) and machine-learning approaches show promise for enhancing prediction and personalising prevention. These models can identify complex patterns in large datasets and may outperform traditional tools, although transparent reporting and robust validation across diverse populations remain essential [66].

Digital health technologies are reshaping intervention delivery through mobile health applications, wearable devices and telemedicine platforms. These tools enable real-time feedback, remote monitoring and extended access to prevention services, especially in underserved areas. However, variable data accuracy, limited regulatory oversight and digital inequities remain important barriers to wider adoption [66].

Advanced imaging techniques, such as coronary artery calcium (CAC) scoring, are providing new insights into subclinical disease and improving risk stratification for intermediate-risk individuals (65). Precision medicine approaches integrating genetic information, polygenic risk scores and novel biomarkers – including lipoprotein(a), inflammatory and proteomic markers – further expand opportunities for personalised prevention, though cost, validation and equity issues limit broad implementation [66].

### Implementation science and systematic approaches

The persistent gap between evidence and practice in cardiovascular prevention underscores the importance of implementation science for translating research into routine clinical and community practice. This discipline offers frameworks, theories and models to identify context-specific barriers and to guide systematic adoption, adaptation and sustainment of effective interventions; recent work in the European Journal of Cardiovascular Nursing has emphasised the need for structured knowledge translation that goes beyond evidence generation and into everyday practice.

Barriers to implementation operate at multiple, interacting levels. At the health-system level, many systems remain organised around acute care, with reimbursement and funding structures that favour procedures and episodic interventions over time-intensive prevention activities; lack of dedicated funding, weak incentives and fragmented care pathways continue to undermine integration of prevention into routine cardiovascular services [67]. Provider-level obstacles include gaps in knowledge and training (particularly in lifestyle medicine and behaviour-change techniques), time pressures that limit counselling, therapeutic inertia, and workforce shortages that reduce capacity for prevention and education. Patient-level factors – such as limited adherence, socioeconomic constraints, cultural influences and low health literacy – further constrain uptake and effectiveness. Organizational challenges include absence of systematic prevention protocols, inadequate quality-measurement and improvement systems, and poor integration across settings, which together limit consistent delivery of comprehensive prevention to all eligible patients.

Despite these challenges, well-designed, multidisciplinary programmes demonstrate that systematic implementation is achievable. Examples such as EUROACTION and GOSPEL achieved significant improvements in risk factors and outcomes through coordinated, team-based approaches and explicit implementation planning [68,69]. Models that support prevention delivery include prevention-focused care pathways with dedicated personnel, Patient-Centered Medical Home and accountable-care arrangements that create accountability for population health, and team-based care that shares prevention responsibilities across pharmacists, nurses, dietitians and other professionals.

Implementation can be further supported by quality-improvement methods that combine measurement, targeted interventions and continuous monitoring to drive sustained change [70, 71]. Taken together, these approaches illustrate that combining implementation science frameworks with practical system-level reforms and team-based service models offers a realistic route to closing the gap between evidence and routine cardiovascular prevention.

### Economic considerations and value-based care

The economic case for cardiovascular prevention is increasingly persuasive as health systems face rising costs and ageing populations; CVD already imposes very large direct healthcare costs in many regions (running into the hundreds of billions annually in high-income settings) [72]. Economic analyses show that effective prevention programmes can deliver favourable returns on investment by reducing healthcare utilisation and improving productivity [73]. Thus, programme costs should be assessed in the context of the total economic burden of CVD, which comprises both direct medical expenditures and substantial indirect costs from lost productivity [72].

Cost-effectiveness evidence is generally supportive, especially for interventions targeted at high-risk groups; systematic reviews recommend prioritising measures that yield the greatest clinical benefit per unit cost [74]. Community-based programmes often provide excellent value because they can reach large populations at relatively modest *per capita* cost, whereas clinical prevention programmes – though more expensive per participant – tend to be cost-effective when targeted at individuals with elevated cardiovascular risk [75].

The wider adoption of value-based care models creates additional opportunities to incentivise prevention. Arrangements such as shared-savings contracts, bundled payments (fixed episode payments), and population-health agreements align financial incentives with outcomes and can encourage providers and payers to invest in prevention, since avoided events reduce utilisation and improve financial performance under these models [76].

### Recommendations for future development

Health systems should re-orient toward prevention and make it a core organisational mission, backed by dedicated resources, clear governance and accountability for prevention outcomes. This re-orientation requires systematic protocols for risk assessment and intervention, and better integration of prevention activities across primary care, hospital services and community settings so that prevention is delivered continuously rather than episodically.

Workforce development must be expanded so that clinicians and allied professionals have practical skills in lifestyle medicine and behaviour-change techniques. Training programmes should teach motivational interviewing, goal-setting, self-monitoring and other evidence-based counselling methods, and should promote interprofessional education to enable effective team-based delivery of prevention services.

Digital technologies and data systems should be thoughtfully integrated into routine care to extend reach and support ongoing monitoring. Electronic health records should enable prevention workflows, validated mobile apps and wearables can support patient engagement and remote follow-up, and telemedicine and remote monitoring should be used to improve access – provided these tools are interoperable, pragmatically validated and embedded into clinical pathways.

Robust quality-measurement and continuous-improvement systems are needed to ensure programmes deliver intended benefits. Routine metrics for prevention processes and outcomes, combined with audit-and-feedback cycles and targeted quality-improvement activities, will help identify performance gaps, test changes and sustain improvements over time.

Prevention will also require stronger community and multisectoral partnerships to address social and environmental determinants of cardiovascular risk that healthcare alone cannot change. Collaborations with schools, workplaces, community organisations and policymakers can create healthier environments and support population-level interventions that complement clinical care.

Research priorities should emphasise implementation science and pragmatic evaluation. Studies must address which delivery models work in which contexts, how to improve patient adherence and engagement, and how to maintain effectiveness and equity at scale; hybrid effectiveness – implementation trials and real-world evaluations are particularly valuable.

Finally, policy and financing reforms are essential to create a supportive environment for prevention. Payment models and reimbursement policies should adequately compensate prevention activities, while regulatory and public-health measures should improve access to healthy foods and safe spaces for physical activity; value-based contracts and other incentives can help align provider payments with population-health outcomes.

**FIGURE 1:**
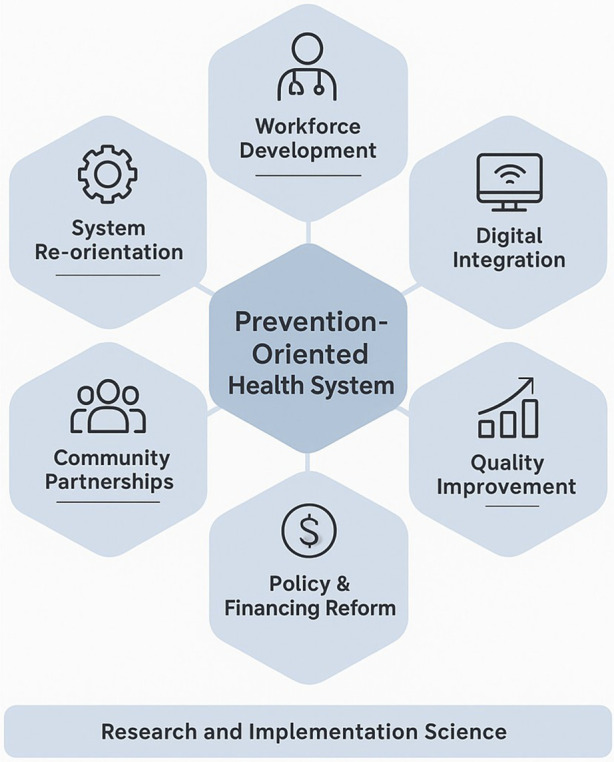
Framework for future development in prevention-oriented health systems.

Taken together, these actions – coordinated and adapted to local context – can help move proven prevention strategies from pilots into routine, equitable and sustainable practice. These interconnected recommendations are summarised in Figure 1, which illustrates the key domains required for developing prevention-oriented health systems.

To make the call to action concrete and measurable, Table 3 presents a minimum indicator set and brief implementation packages stratified by level of action.

**TABLE 3:** Minimum call-to-action indicator set and implementation packages, stratified by level of action.

Level	What to do	Who does it	How progress is measured
Policy/Financing	Introduce or expand value-based payment for prevention; mandate systematic CVD risk screening in primary care	Health ministries; payers; national health authorities	% Of eligible patients with documented CVD risk assessment; prevention spend as % of health budget
Service organisation	Implement automatic referral to cardiac rehabilitation; establish structured lifestyle-change clinics	Hospital managers; cardiology departments; primary care leads	Cardiac rehabilitation referral rate (%); % patients meeting LDL-C and BP targets at 12 months
Workforce	Train nurses, pharmacists, and allied health professionals in lifestyle medicine and behaviour-change counselling	Professional bodies; universities; continuing education providers	% Of prevention staff with certified lifestyle medicine training; patient-reported counselling quality scores
Patient/Community	Co-design prevention programmes with patients and communities; deploy peer-support networks	Community health organisations; patient groups; local government	Patient activation measure scores; programme completion rate; population-level risk factor trends

### Limitations

This paper provides a structured overview and discussion rather than a systematic review. As such, the selection of evidence may not be exhaustive, and the synthesis reflects the authors’ judgement of the most relevant and influential sources. Heterogeneity in interventions, populations and outcomes limited opportunities for quantitative pooling and precluded comprehensive meta-analysis across programme types. Publication bias and variable reporting quality in primary studies may have influenced reported effects. Generalisability of findings across different health-system contexts – particularly in low- and middle-income countries – may be limited, and economic and implementation evidence is often context specific. Finally, evidence for emerging technologies and new models of care is still evolving, with limited long-term effectiveness or cost-effectiveness data, underscoring the need for pragmatic real-world evaluation.

As a narrative review, this paper is subject to the inherent limitations of that design. The absence of a pre-registered protocol, exhaustive database search, or formal quality appraisal of included studies means that the synthesis may not capture the full body of evidence and is susceptible to selection bias. To reduce this risk, the evidence presented was drawn primarily from high-quality systematic reviews, major clinical guidelines, and large multicentre registries. Readers should interpret the narrative synthesis with appropriate caution and refer to primary sources and systematic reviews for definitive evidence on specific interventions.

### Conclusion

Evidence from both community-based and clinical cardiovascular prevention programmes demonstrates clear and growing benefits in reducing population risk and improving individual outcomes. However, implementation remains limited by multilevel barriers spanning health systems, organisations, and patient engagement. Emerging technologies – including digital tools, advanced risk models, and AI – offer new opportunities to enhance prevention and streamline delivery, but require careful validation and integration into practice. Sustained, equitable impact will depend on coordinated efforts across healthcare, policy, and community sectors to scale proven interventions and embed prevention throughout the life course.
